# Supplementary material for: Biochemical and structural characterization of the BioZ enzyme engaged in bacterial biotin synthesis pathway
Source: Nat Commun. 2021 Apr 6;12:2056. doi: 10.1038/s41467-021-22360-4 (PMC8024396; doi:10.1038/s41467-021-22360-4)
Supplement: Supplementary file 1 — Supplementary Information [file 41467_2021_22360_MOESM1_ESM.pdf]

Supporting information

Supplementary Tables

Supplementary Table 1 Bacteria and plasmids used in this study

Strains or plasmids	Relevant characteristics	Origins
Strains		
DH5α	A cloning host of *E. coli*	Lab stock
BL21(DE3)	An expression host of *E. coli*	Lab stock
MG1655	A wild-type strain of *E. coli*	Lab stock
NTL4	A tetracycline-sensitive strain of *Agrobacterium tumefaciens* C58	Lab stock, [1]
FYJ283	A biotin auxotrophic strain of *Agrobacterium tumefaciens* NTL4 (Δ*bioBFDA*)	Lab stock, [1]
ER90	A biotin auxotrophic strain of MG1655 (Δ*bioF*/Δ*bioF*/Δ*bio*D)	Lab stock, [2]
STL24	MG1655, Δ*bioH*	Lab stock, [2]
STL96	MG1655, Δ*bioC*	Lab stock, [2]
FYJ391	ST24(MG1655, Δ*bioH*) carrying pBAD322	Lab stock, [2]
FYJ415	STL96(MG1655, Δ*bioC*) carrying pBAD322	Lab stock, [2]
FYJ428	BL21(DE3) carrying pET28a::*bioZ*	This work
FYJ1661	ST24(MG1655, Δ*bioH*) carrying pBAD24::*bioZ*	This work
FYJ1662	STL96(MG1655, Δ*bioC*) carrying pBAD24::*bioZ*	This work
FYJ1622	STL24(MG1655, Δ*bioH*) carrying pBAD24::*bioZ*(C115A)	This work
FYJ1623	STL24(MG1655, Δ*bioH*) carrying pBAD24::*bioZ*(H255A)	This work
FYJ1624	STL24(MG1655, Δ*bioH*) carrying pBAD24::*bioZ*(N285A)	This work
FYJ1625	STL24(MG1655, Δ*bioH*) carrying pBAD24::*bioZ*(R39A)	This work
FYJ1626	STL24(MG1655, Δ*bioH*) carrying pBAD24::*bioZ*(R153A)	This work
FYJ1627	STL24(MG1655, Δ*bioH*) carrying pBAD24::*bioZ*(R221A)	This work
FYJ1628	STL24(MG1655, Δ*bioH*) carrying pBAD24::*bioZ*(R260A)	This work
FYJ1629	STL24(MG1655, Δ*bioH*) carrying pBAD24::*bioZ*(K39A/K153A)	This work
FYJ1630	STL24(MG1655, Δ*bioH*) carrying pBAD24::*bioZ*(K39A/R221A)	This work
FYJ1631	STL24(MG1655, Δ*bioH*) carrying pBAD24::*bioZ*(K39A/R260A)	This work
FYJ1632	STL24(MG1655, Δ*bioH*) carrying	This work

	pBAD24::*bioZ*(K153A/K221A)	
FYJ1633	STL24(MG1655, Δ*bioH*) carrying pBAD24::*bioZ*(K153A/R260A)	This work
FYJ1634	STL24(MG1655, Δ*bioH*) carrying pBAD24::*bioZ*(K221A/R260A)	This work
FYJ1635	STL24(MG1655, Δ*bioH*) carrying pBAD24::*bioZ*(R39A/R153A/R221A)	This work
FYJ1636	STL24(MG1655, Δ*bioH*) carrying pBAD24::*bioZ*(R39A/R153A/R260A)	This work
FYJ1637	STL24(MG1655, Δ*bioH*) carrying pBAD24::*bioZ*(R39A/R221A/R260A)	This work
FYJ1638	STL24(MG1655, Δ*bioH*) carrying pBAD24::*bioZ*(R153A/R221A/R260A)	This work
FYJ1639	STL24(MG1655, Δ*bioH*) carrying pBAD24::*bioZ*(R39A/R153A/R221A/R260A)	This work
FYJ1640	STL96(MG1655, Δ*bioC*) carrying pBAD24-*bioZ*(C115A)	This work
FYJ1641	STL96(MG1655, Δ*bioC*) carrying pBAD24::*bioZ*(H255A)	This work
FYJ1642	STL96(MG1655, Δ*bioC*) carrying pBAD24::*bioZ*(N285A)	This work
FYJ1643	STL96(MG1655, Δ*bioC*) carrying pBAD24::*bioZ*(R39A)	This work
FYJ1644	STL96(MG1655, Δ*bioC*) carrying pBAD24::*bioZ*(R153A)	This work
FYJ1645	STL96(MG1655, Δ*bioC*) carrying pBAD24::*bioZ*(R221A)	This work
FYJ1646	STL96(MG1655, Δ*bioC*) carrying pBAD24::*bioZ*(R260A)	This work
FYJ1647	STL96(MG1655, Δ*bioC*) carrying pBAD24::*bioZ*(K39A/K153A)	This work
FYJ1648	STL96(MG1655, Δ*bioC*) carrying pBAD24::*bioZ*(K39A/R221A)	This work
FYJ1649	STL96(MG1655, Δ*bioC*) carrying pBAD24::*bioZ*(K39A/R260A)	This work
FYJ1650	STL96(MG1655, Δ*bioC*) carrying pBAD24::*bioZ*(K153A/K221A)	This work
FYJ1651	STL96(MG1655, Δ*bioC*) carrying pBAD24::*bioZ*(K153A/R260A)	This work
FYJ1652	STL96(MG1655, Δ*bioC*) carrying pBAD24::*bioZ*(K221A/R260A)	This work
FYJ1653	STL96(MG1655, Δ*bioC*) carrying pBAD24::*bioZ*(R39A/R153A/R221A)	This work
FYJ1654	STL96(MG1655, Δ*bioC*) carrying	This work

	pBAD24::*bioZ*(R39A/R153A/R260A)	
FYJ1655	STL96(MG1655, Δ*bioC*) carrying pBAD24::*bioZ*(R39A/R221A/R260A)	This work
FYJ1656	STL96(MG1655, Δ*bioC*) carrying pBAD24::*bioZ*(R153A/R221A/R260A)	This work
FYJ1657	STL96(MG1655, Δ*bioC*) carrying pBAD24::*bioZ*(R39A/R153A/R221A/R260A)	This work
FYJ4001	STL24(MG1655, Δ*bioH*) carrying pBAD24::*bioZ*(S84A)	This work
FYJ4002	STL24(MG1655, Δ*bioH*) carrying pBAD24::*bioZ*(R147A)	This work
FYJ4003	STL24(MG1655, Δ*bioH*) carrying pBAD24::*bioZ*(S287A)	This work
FYJ4004	BL21(DE3) carrying pET28a::At*fabH1*	This work
FYJ4005	BL21(DE3) carrying pET28a::Ec*fabH*	This work
Plasmids		
pBAD24	An arabinose-inducible expression vector, Amp^R	Lab stock
pET28a	a T7 promoter-driven expression vector, Km^R	Novagen
pBAD24::*bioZ*	pBAD24 encoding *bioZ*, Amp^R	Lab stock
pET28a::*bioZ*	pET28a encoding *bioZ*, Km^R	Lab stock
pBAD24::*bioZ*(C115A)	pBAD24 encoding the mutant version of *bioZ* (C115A), Amp^R	This work
pBAD24::*bioZ*(H255A)	pBAD24 encoding the mutant version of *bioZ* (H255A), Amp^R	This work
pBAD24::*bioZ*(N285A)	pBAD24 encoding the mutant version of *bioZ*(N285A), Amp^R	This work
pBAD24::*bioZ*(R39A)	pBAD24 encoding the mutant version of *bioZ* (R39A), Amp^R	This work
pBAD24::*bioZ*(R153A)	pBAD24 encoding the mutant version of *bioZ* (R153A), Amp^R	This work
pBAD24::*bioZ*(R221A)	pBAD24 encoding the mutant version of *bioZ* (R221A), Amp^R	This work
pBAD24::*bioZ*(R260A)	pBAD24 encoding the mutant version of *bioZ* (R260A), Amp^R	This work
pBAD24::*bioZ*(R39A/R153A)	pBAD24 encoding the mutant version of *bioZ* (R39A/R153A), Amp^R	This work
pBAD24::*bioZ* (R39A/R221A)	pBAD24 encoding the mutant version of *bioZ* (R39A/R221A), Amp^R	This work
pBAD24::*bioZ* (R39A/R260A)	pBAD24 encoding the mutant version of *bioZ* (R39A/R260A), Amp^R	This work
pBAD24::*bioZ* (R153A/R221A)	pBAD24 encoding the mutant version of *bioZ* (R153A/R221A), Amp^R	This work

pBAD24::*bioZ* (R153A/R260A)	pBAD24 encoding the mutant version of *bioZ* (R153A/R260A), Amp^R	This work
pBAD24::*bioZ* (R221A-R260A)	pBAD24 encoding the mutant version of *bioZ* (R221A/R260A), Amp^R	This work
pBAD24::*bioZ* (R39A/R153A/R221A)	pBAD24 encoding the mutant version of *bioZ* (R39A/R153A/R221A), Amp^R	This work
pBAD24::*bioZ* (R39A/R153A/R260A)	pBAD24 encoding the mutant version of *bioZ* (R39A/R153A/R260A), Amp^R	This work
pBAD24::*bioZ* (R39A/R221A/R260A)	pBAD24 encoding the mutant version of *bioZ* (R39A/R221A/R260A), Amp^R	This work
pBAD24::*bioZ* (R153A/R221A/R260A)	pBAD24 encoding the mutant version of *bioZ* (R153A/R221A/R260A), Amp^R	This work
pBAD24::*bioZ* (R39A/R153A/R221A/R260A)	pBAD24 encoding the mutant version of *bioZ* (R39A/R153A/R221A/R260A), Amp^R	This work
pBAD24::*bioZ*(S84A)	pBAD24 encoding the mutant version of *bioZ* (S84A), Amp^R	This work
pBAD24::*bioZ*(R147A)	pBAD24 encoding the mutant version of *bioZ* (R147A), Amp^R	This work
pBAD24::*bioZ*(S287A)	pBAD24 encoding the mutant version of *bioZ* (S287A), Amp^R	This work
pET28a::At*fabH*	pET28a encoding *fabH from A. tumefaciens*, Km^R	This work
pET28a::Ec*fabH*	pET28a encoding *fabH from E. coli*, Km^R	This work

Supplementary Table 2 Primers used in this study

**Primers**	**Sequences**
AtFabH-F	5’-GCG GGA TCC ATG ATC CGC TCT ATA GTC CG-3’
AtFabH-R	5’-CCG CTC GAG TTA CCA GCG CAG CAG CAC TG -3’
EcFabH-F	5’-GGG AAT TCC ATA TGT ATA CGA AGA TTA TTG GTA CTG GCA GC-3’
EcFabH-R	5’-CCG CTC GAG GAA ACG AAC CAG CGC GGA G-3’
bioZ(C115A)-F	5’-C**GC A**TC CGG GTT TCT TTA TGC GCT TAC CCT CG-3’
bioZ(C115A)-R	5’-AAA GAA ACC CGG ATG CGG CCC CGG CAA GAT CGA T-3’
bioZ(H255A)-F	5’-TGC CA**G CA**C AGG CCA ATG CCC GCA TGT CCG AC-3’
bioZ(H255A)-R	5’-ATT GGC CTG **TGC** TGG CAC GAA ACG GCT GAT AT-3’
bioZ(N285A)-F	5’-AAG CTT CGG C**GC A**TC TTC CGC TGC TAC CAT CCC-3’
bioZ(N285A)-R	5’-AAG A**TG C**GC CGA AGC TTC CTA TGG TGC GGA CC-3’
bioZ-UP-F	5’-TGG GCT AGC AGG AGG AAT TCA TGC AGA CAC GTT CTT CCC GC-3’
bioZ(R39A)-R	5’-GAA TGC CGG T**TG C**TC GCT CGA TCC ACC CGG CT-3’
bioZ(R39A)-F	5’-CGA GCG A**GC A**AC CGG CAT TCG CTC CCG TTA T-3’
bioZ(R153A)-R	5’-CGG CGC TCG C**TG C**TT CCG CCG GAT TGA TGC GGC-3’
bioZ(R153A)-F	5’-GGC GGA A**GC A**GC GAG CGC CGT CCT GTT TGC-3’
bioZ(R221A)-R	5’-AGA AAA CCT C**TG C**GC CAT CGC GCA TCG TCA TCA-3’
bioZ(R221A)-F	5’-CGA TGG C**GC A**GA GGT TTT CTC TCG CGC CGT-3’
bioZ(R260A)-R	5’-CGT CGG ACA T**TG C**GG CAT TGG CCT GAT GTG GCA-3’
bioZ(R260A)-F	5’-CAA TGC C**GC A**AT GTC CGA CGC CGT TTG CGG-3’
bioZ-Down-R	5’-TGG TGG TGG TGG TGG TCG ACG ACG CGA TAA ACC ACC GCC C-3’
bioZ(S84A)-F	5’-**GCA** ACG CCG GAT CAT CTT CTG CCG CCT TCC GC-3’
bioZ(S84A)-R	5’-AGA TGA TCC GGC GT**T GC**G GTG GCA AGC AGC GTC AG-3’
bioZ(R147A)-F	5’-TCA GCC GC**G CA**A TCA ATC CGG CGG AAA GGG CG-3’
bioZ(R147A)-R	5’-ATT GAT **TGC** GCG GCT GAG AAT ATT GGC GGC GA-3’
bioZ(S287A)-F	5’-CAA TTC T**GC A**GC TGC TAC CAT CCC GCT TTC GT-3’
bioZ(S287A)-R	5’-TAG CAG C**TG C**AG AAT TGC CGA AGC TTC CTA TG -3’

The codons with a point mutation are indicated with bold letters.

Supplementary Figures

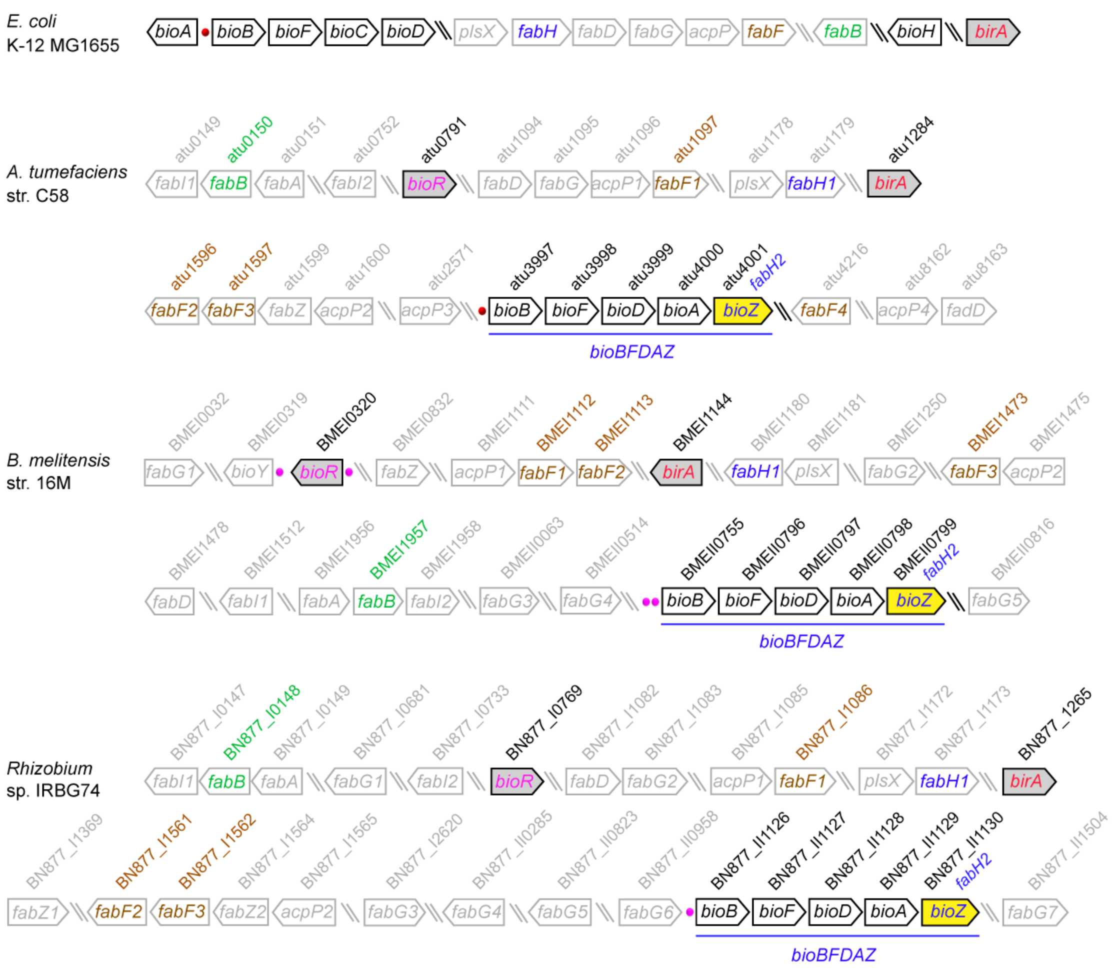

Supplementary Figure 1 Genomic context of fatty acid (and biotin) biosynthesis operon

Namely, the reference sequences of four different genomes derive from *E. coli* K-12 MG1655 (Acc. no.: NC_000913), *A. tumefaciens* str. C58 (Acc. no.: NC_003062 and NC_003063), *B. melitensis* str. 16M (Acc. no.: NC_003317 and NC_003318), *Rhizobium* sp. IRBG74 (Acc. no.: NC_022535 and NC_022536).

Red dot denotes the cognate DNA sequences recognized by BirA, and pink dots represent the BioR signals. It is likely that gene duplication and amplification is prevalent in the context of fatty acid synthesis. *fabH2*, the duplicated gene of *fabH1*, is well domesticated because that it is integrated into a unique *bioBFDAZ* operon restricted to α-proteobacteria lacking "*bioH* and *bioC*". Thus, *fabH2* is renamed *bioZ* (highlighted with yellow background).

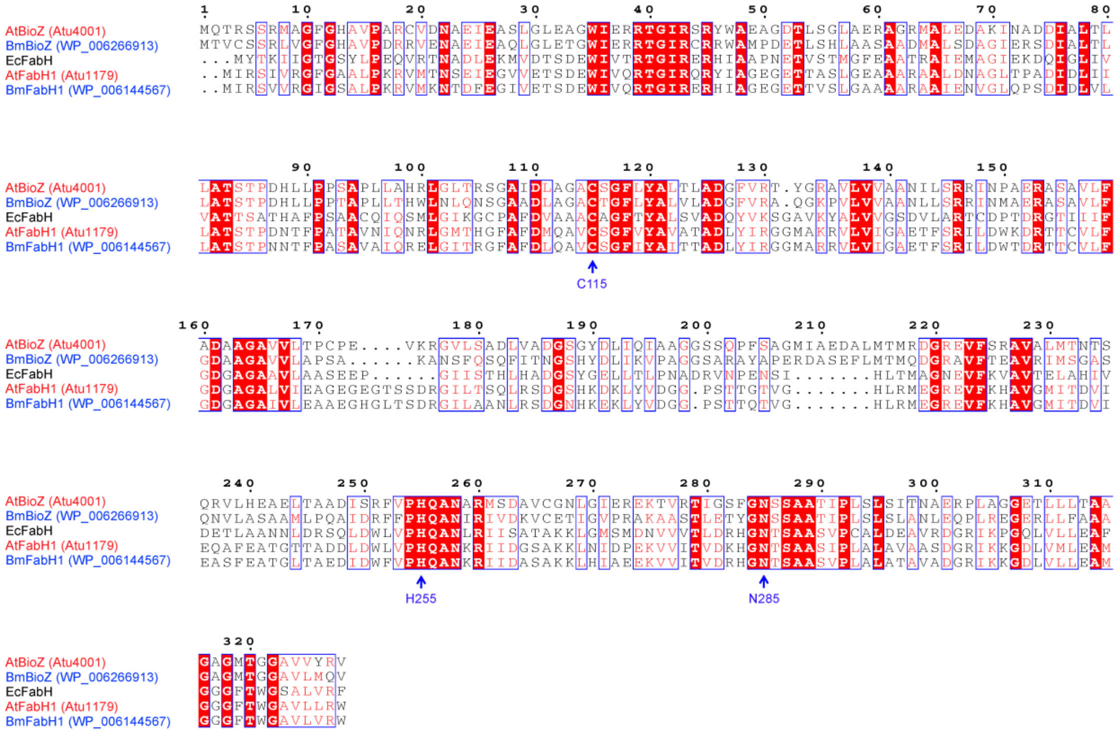

Supplementary Figure 2 Bioinformatic analysis of FabH and its BioZ homologue

Three conserved sites of catalytic triad are indicated with blue arrows. Multiple sequence alignment was conducted with Clustal Omega (https://www.ebi.ac.uk/Tools/msa/clustalo).

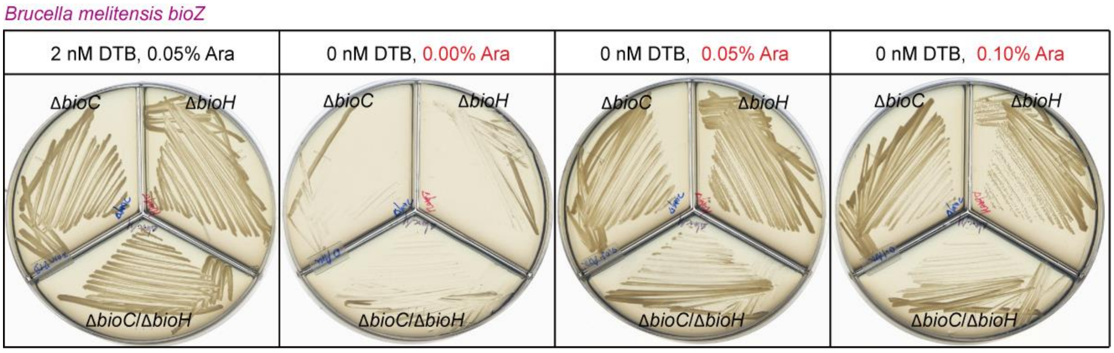

Supplementary Figure 3 The *Brucella bioZ* can complement the single Δ*bioC* (and/or Δ*bioH*) mutant and the double mutant (Δ*bioC/*Δ*bioH*) of *E. coli* on the non-permissive growth condition

The *E. coli* strains were plated on the M9 minimal agar media, and then maintained at 30°C overnight.

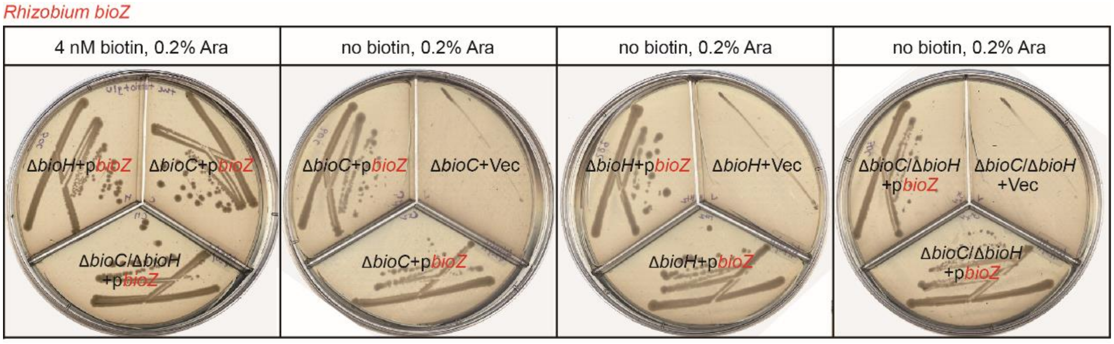

Supplementary Figure 4 The *Rhizobium BioZ* restores bacterial growth of the Δ*bioC* (and/or Δ*bioH*) single mutant and the double mutant (Δ*bioC/*Δ*bioH*) of *E. coli* on the biotin-deficient condition

The engineered *E. coli* strains were inoculated on the M9 minimal agar plates, and then kept at 30°C overnight.

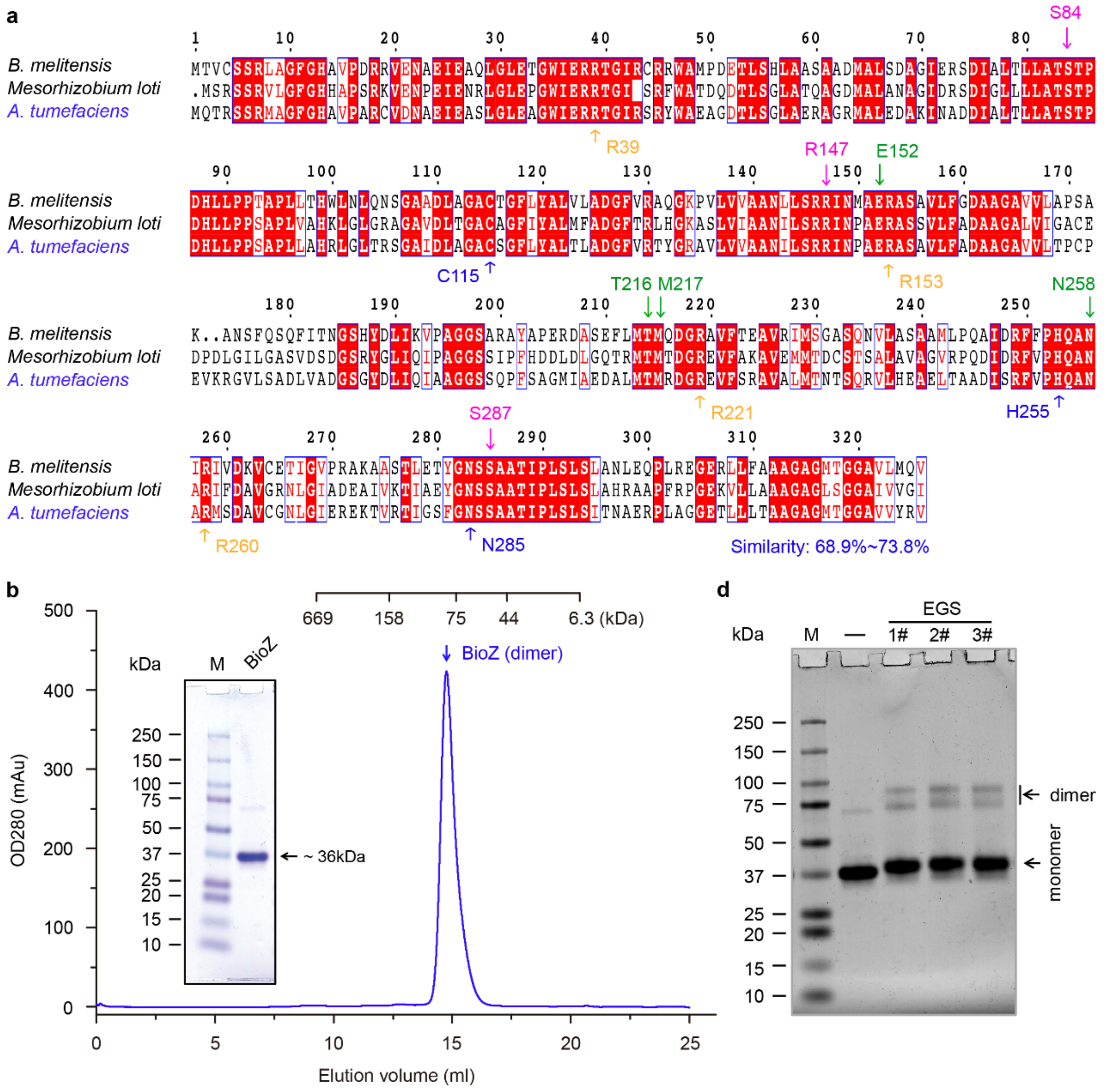

c

1 MQTR**SSRMAG FGHAVPAR**CV DNAEIEASLG LEAGWIER**RT GIRSRYWAEA**

51 **GDTLSGLAER AGRMALEDAK INADDIALTL LATSTPDHLL PPSAPLLAHR**

101 LGLTRSGAID LAGACSGFLY ALTLADGFVR TYGRAVLVVA ANILSR**RINP**

151 **AER**ASAVLFA DAAGAVVLTP CPEVKRGVLS ADLVADGSGY DLIQIAAGGS

201 SQPF**SAGMIA EDALMTMRDG REVFSRAVAL MTNTSQRVLH EAELTAADIS**

251 **R**FVPHQANAR **MSDAVCGNLG IEREK**TVRTI GSFGNSSAAT IPLSLSITNA

301 ERPLAGGETL LLTAAGAGMT GGAVVYRV

Coverage: 44.2%

Supplementary Figure 5 Characterization of the *A. tumefaciens* BioZ protein
a. Sequence analysis of BioZ homologs from *A. tumefaciens*, *B. melitensis*, and *Mesorhizobium loti*

Among them, the similarity varied from 68.9% to 73.8%, and critical residues of catalytic triad are labeled.

The residues of catalytic triad are indicated with blue arrows; the ACP-

interacting residues are colored orange; the putative residues that neutralize the free carboxyl group of glutaryl-CoA are labeled with pink arrows; and the proposed sites that stabilize CoA cargo are colored green.

b. Size exclusion analysis of the purified AtBioZ protein

The concentrated protein of AtBioZ (~500μl) was loaded on the column of Superdex 200 increase (GE Healthcare). The insert SDS-PAGE (20% gradient gel) is used to judge the purity of the collected AtBioZ protein from the peak of gel filtration indicated with an arrow. The elution volume of AtBioZ (~15ml) suggests its dimeric form.

c. MS identity of the digested peptides from AtBioZ protein
The matched peptides are shown in red and bold letters, and the whole coverage is 44.2%.

d. Use of chemical cross-linking assay to probe the dimeric manner of AtBioZ protein
The chemical cross-linker refers to EGS, i.e., ethylene glycol bis (succinimidyl succinate). The samples of AtBioZ in triplicate are numbered (1#, 2#, and 3#).

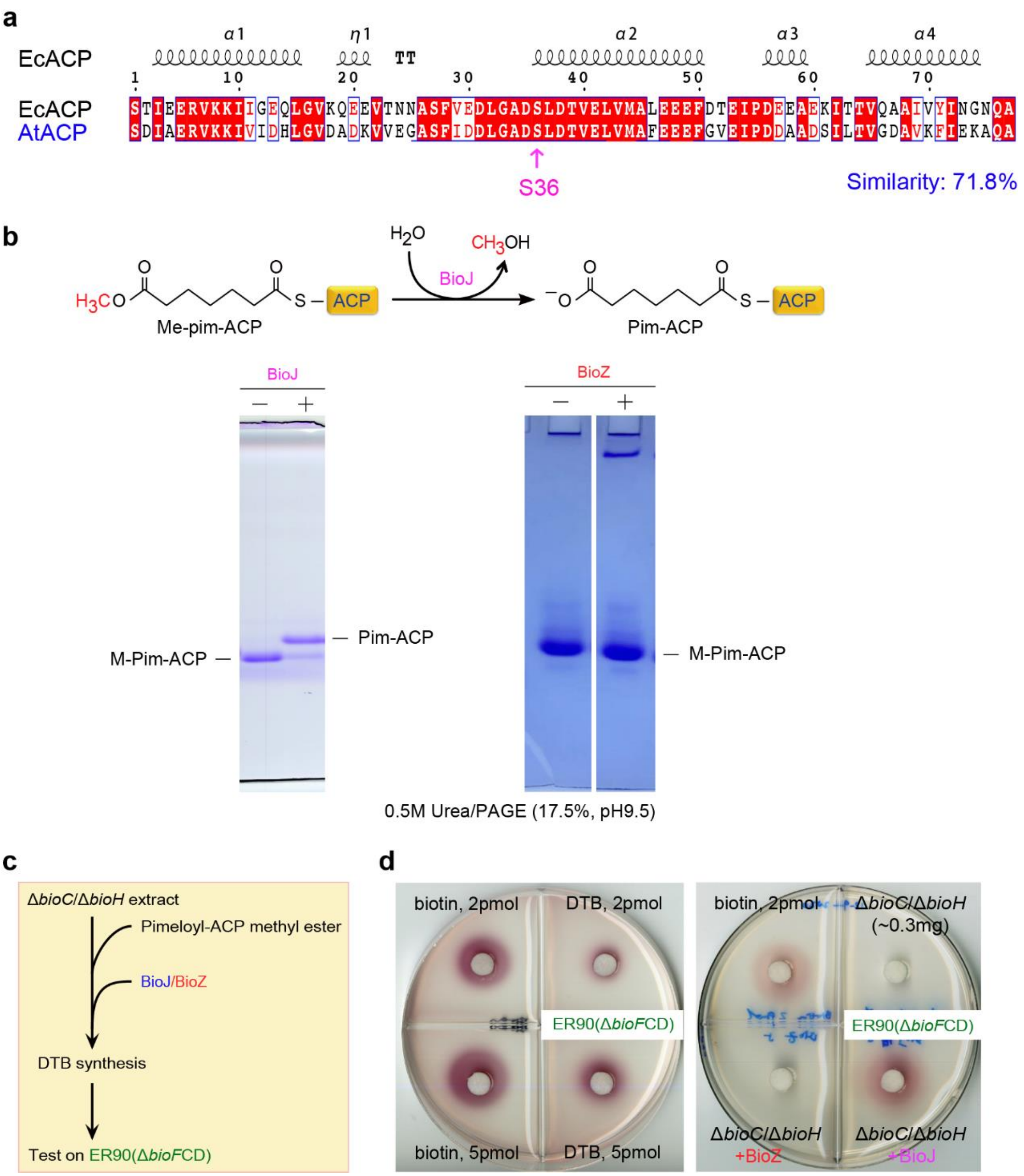

Supplementary Figure 6 BioZ does not behave as BioJ in the DTB/biotin bioassay

a. Sequence alignment of the ACP proteins of *E. coli* and *A. tumefaciens*
The residue Ser 36(S36) denotes the conserved biotinylation site of ACP species.

b. Unlike BioJ, BioZ cannot remove the methyl moiety of pimeloyl-ACP methyl ester
A representative gel is given from 3 different experiments.

c. Scheme for the DTB/biotin bioassay

d. The addition of BioZ protein does not produce DTB/biotin to allow the growth of the ER90 biotin auxotrophic strain

As the positive control, DTB and biotin were spotted on the paper disc, supporting the growth of an indicator strain ER90. Generally consistent with an earlier observation [3], BioZ does not behave as BioJ does in the biotin bioassay.

Designations: Ec, *E. coli*; At, *A. tumefaciens*; M-Pim-ACP, Methyl-Pimeloyl-ACP; Pim-ACP, Pimeloyl-ACP. “--” denotes no addition of BioJ or BioZ protein.

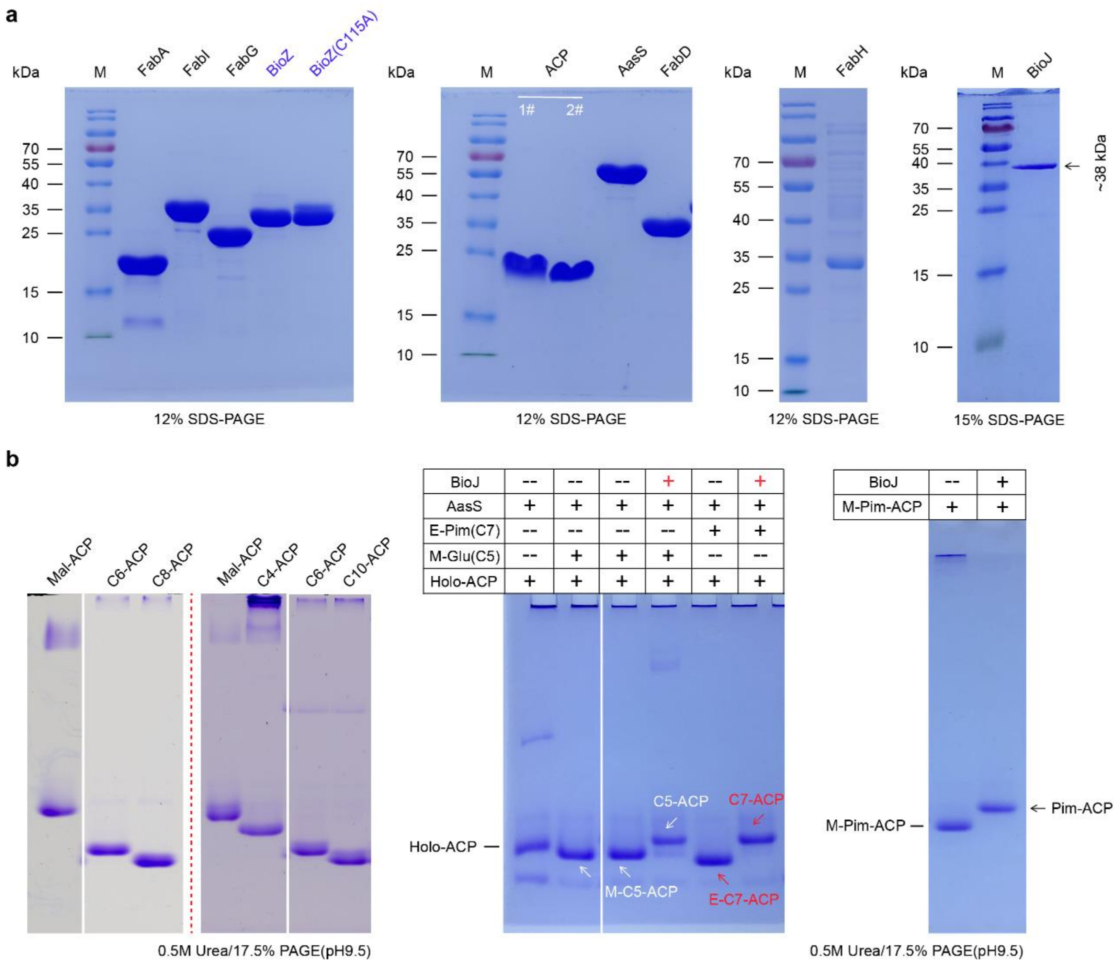

Supplementary Figure 7 The enzymes involved in biosynthesis of fatty acids (and/or biotin precursors)

a. Protein components used for the *in vitro* reconstituted system of bacterial fatty acid synthesis

A number of proteins were separated with the electrophoresis of SDS-PAGE (12% or 15%). The proteins/enzymes used here are listed as follows: FabA, β-hydroxyacyl-ACP dehydratase/isomerase; FabI, enoyl-ACP reductase; FabG, β-ketoacyl ACP reductase; FabH, β-ketoacyl ACP synthases III; BioZ, a second annotated FabH homolog (FabH2) of *A. tumefaciens* [1]; BioZ(C115A), a single mutant of BioZ with the substitution of C115A; ACP, acyl carrier protein; AasS, acyl-ACP synthetase of *Vibrio harveyi* B392 [4]; FabD, Malonyl-CoA: ACP transacylase; BioJ, pimeloyl-ACP methyl ester carboxylesterase of *Francisella novicida* [3,5].

b. Use of conformationally-sensitive electrophoresis to separate a variety of acyl-ACP species

The conformationally-sensitive electrophoresis was performed with 0.5M urea PAGE (17.5%, pH9.5). A representative result was given.

Designations: kDa, kilodaltons; M, Protein marker; Mal-ACP, Malonyl-ACP; C4-ACP: Butyryl-ACP; C6-ACP, Hexanoyl-ACP; C8-ACP, Octanoyl-ACP; C10-ACP, Decanoyl-ACP; M-Glu (C5), Mono-methyl glutaric acid; E-Pim, Mono-ethyl pimeleic acid; M-Pim-ACP, pimeloyl-ACP monomethyl ester.

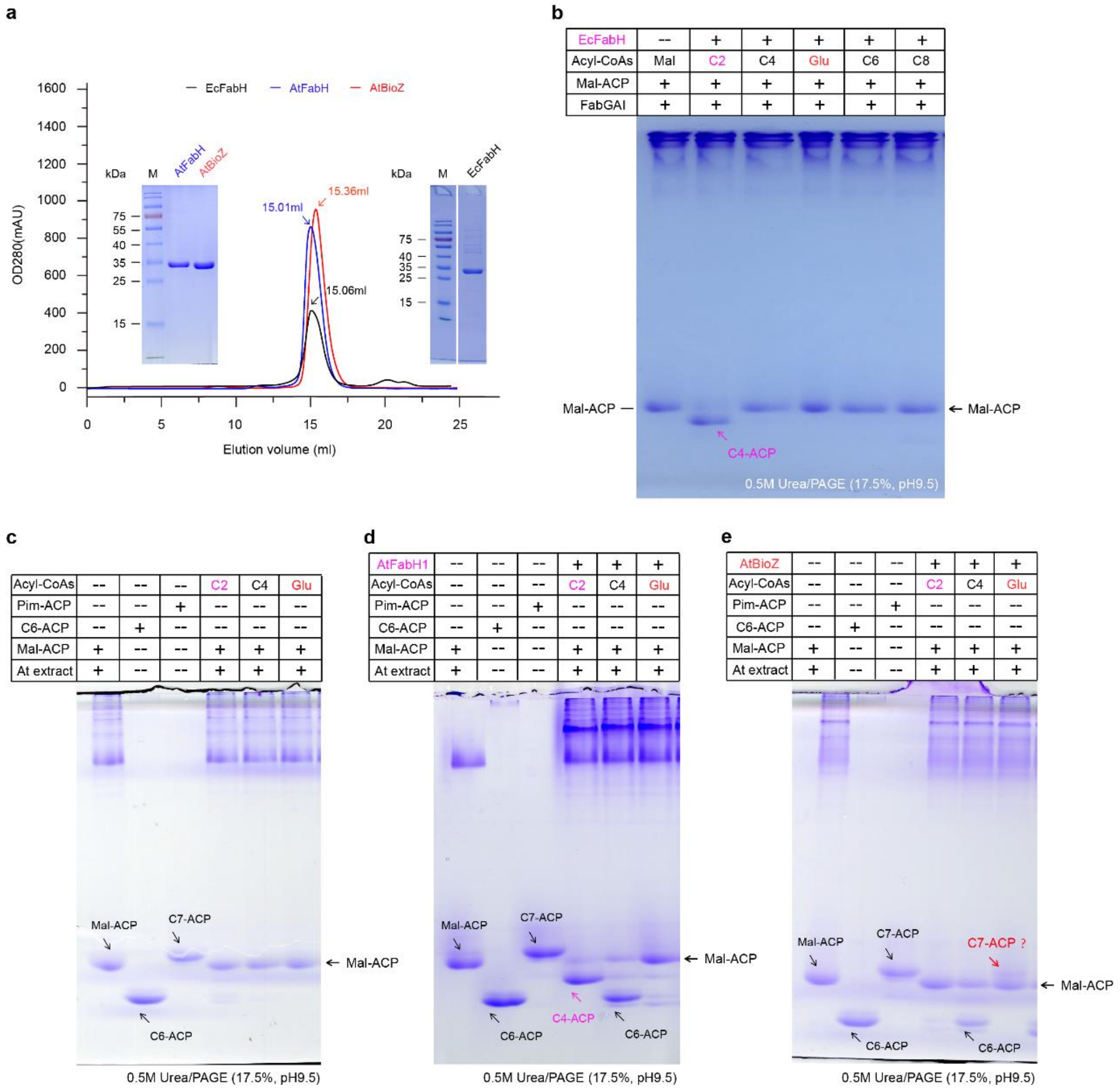

Supplementary Figure 8 Comparison of BioZ and FabH in the selectivity of acyl-CoAs as primer substrates

a. Gel filtration analysis of BioZ enzyme and two different FabH homologs The purity of the FabH and BioZ was verified with the inside gels. Abbreviations: At, *Agrobacterium tumefaciens*; Ec, *Escherichia coli*.

b. The *E. coli* FabH can ligate malonyl-ACP with acetyl-CoA to give butaryl-ACP in the context of successive catalysis of FabG, FabA, and FabI

c. The cell-free crude extract of *A. tumefaciens* (the negative control) did not present the detected activity of condensing malonyl-ACP with a given one of the following three primer substrates, acetyl-CoA (C2-CoA), butyryl-CoA (C4-CoA), and glutaryl-CoA (Glu-CoA)

d. The *A. tumefaciens* FabH1 can recognize the two primer substrates of acetyl-CoA and butyryl-CoA

e. The *Agrobacterium* BioZ can utilize glutaryl-CoA to initiate the ligation with malonyl-ACP, giving keto-pimeloyl-ACP

The reaction mixture of the *in vitro* fatty acid synthesis was separated with an electrophoresis of conformationally-sensitive 0.5M urea/PAGE (17.5%, pH9.5). A representative result of three assays was displayed.

Designation: Mal, Malonyl; C2, Acetyl; C4, Butyryl; Glu, Glutaryl; C6, Hexanoyl; C7, pimeloyl (keto-pimeloyl, hydroxyl-pimeloyl, or enoyl-pimeloyl); C8, Octanoyl; FabGAI, FabG/FabA/FabI; minus, no addition of enzymes or acyl-CoAs; plus, the addition of enzymes or acyl-CoAs.

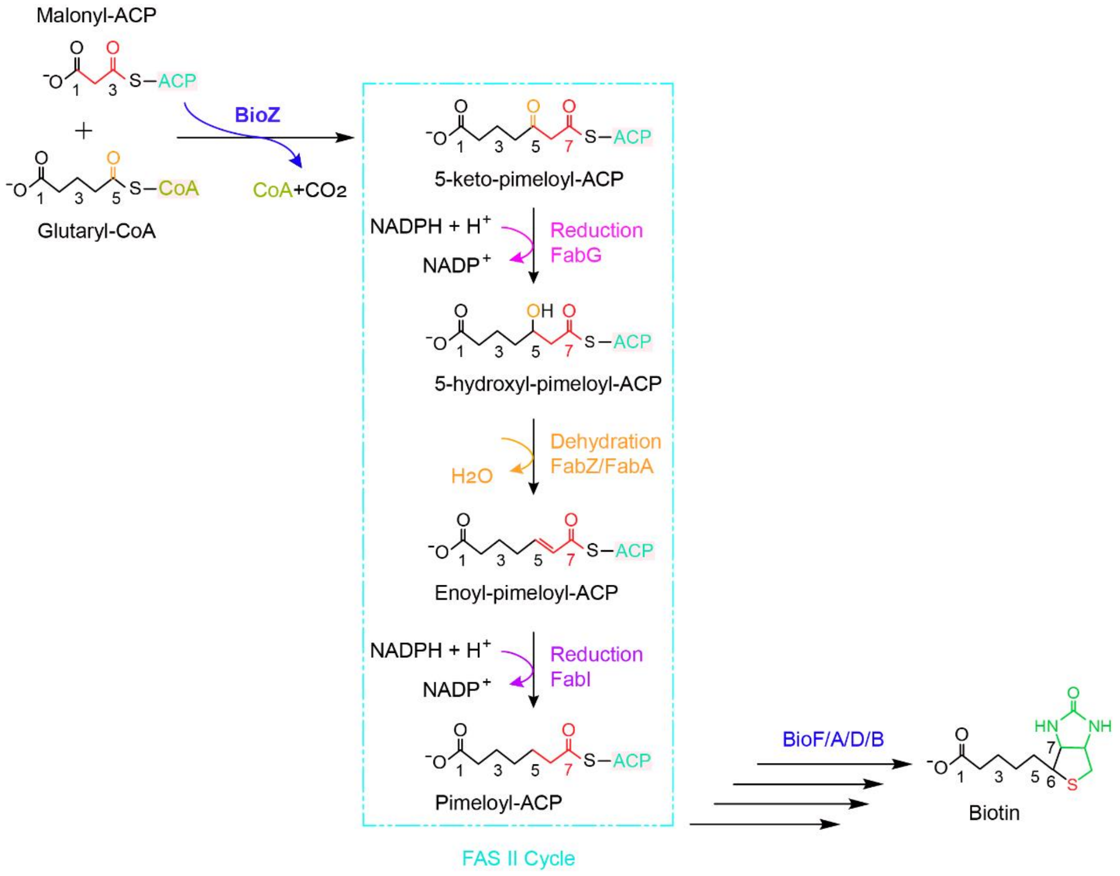

Supplementary Figure 9 A working model for the third biotin synthesis pathway initiated by BioZ

The 5-keto-pimeloyl-ACP product of BioZ reaction proceeds a round of FAS II cycle to give pimeloyl-ACP, which is a precursor for the latter steps of biotin synthesis.

Abbreviations: FAS II, type II path of fatty acid synthesis

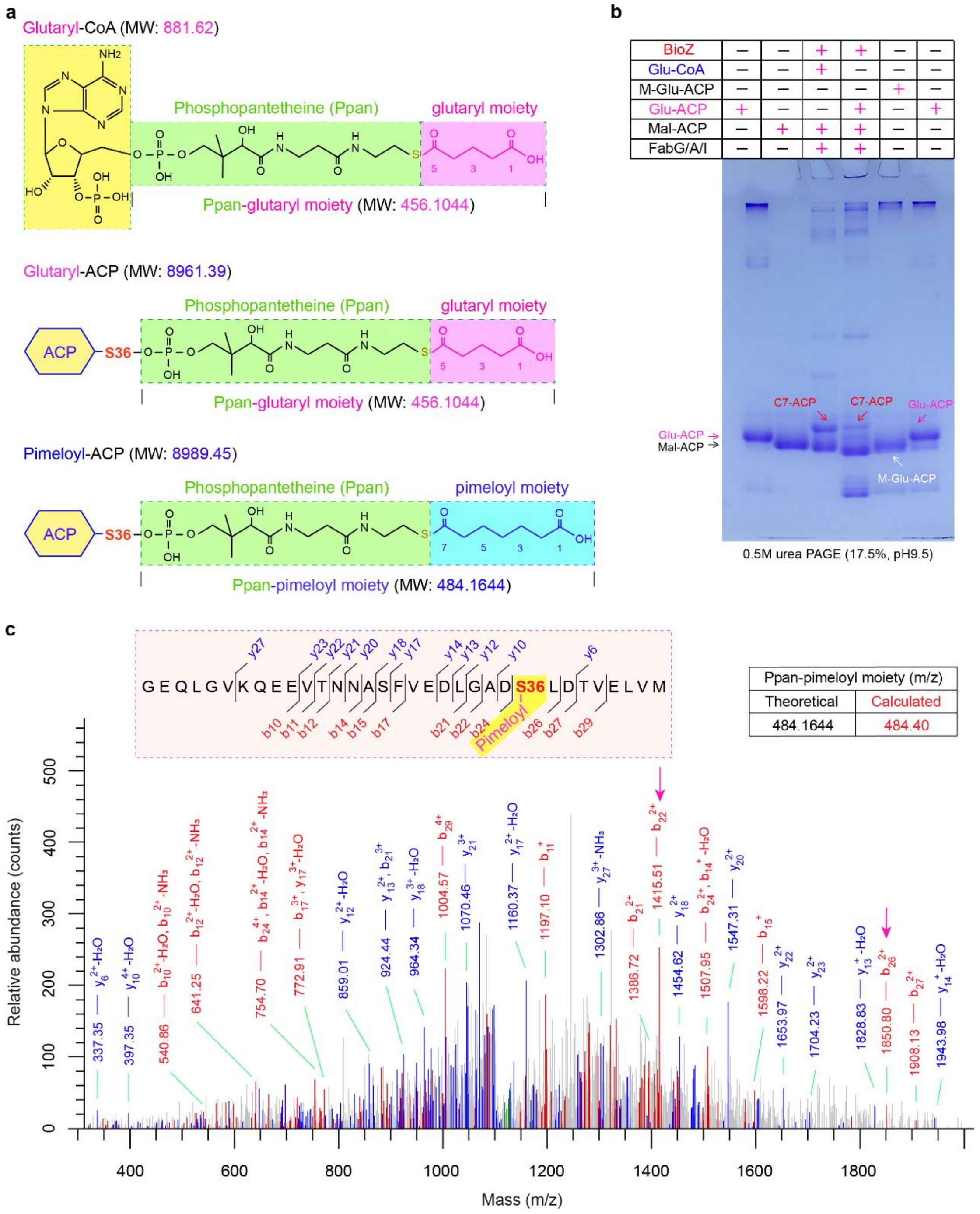

Supplementary Figure 10 MS/MS detection of pimeloyl-ACP product from the *in vitro* BioZ reaction

a. Scheme for chemical structure of the substrate glutaryl-CoA (ACP) and the product pimeloyl-ACP in the proposed BioZ reaction

b. BioZ can catalyze the condensation of glutaryl-CoA (ACP) with malonyl-ACP in the *in vitro* reconstituted reaction of fatty acid synthesis
A representative result was shown from three different trials.
Designations: Ppan, Phosphopantetheine; Mal-ACP, Malonyl-ACP; Glu-CoA

(ACP): Glutaryl-CoA (ACP); M-Glu-ACP, Monomethyl glutaryl-ACP; C7-ACP, pimeloyl-ACP or its unstable intermediates (5-keto-pimeloyl-ACP, 5-hydroxyl-pimeloyl-ACP, and enoyl-pimeloyl-ACP).

c. MS/MS evidence that pimeloyl-ACP is produced in the BioZ reaction

The two peptides indicated with pink arrows were used to determine acyl modification. The mass was calculated to be 484.40, highly close to the theoretical value (484.1644) of Ppan-linked pimeloyl moiety. Therefore, it was reasonable to assign the pimeloyl modification.

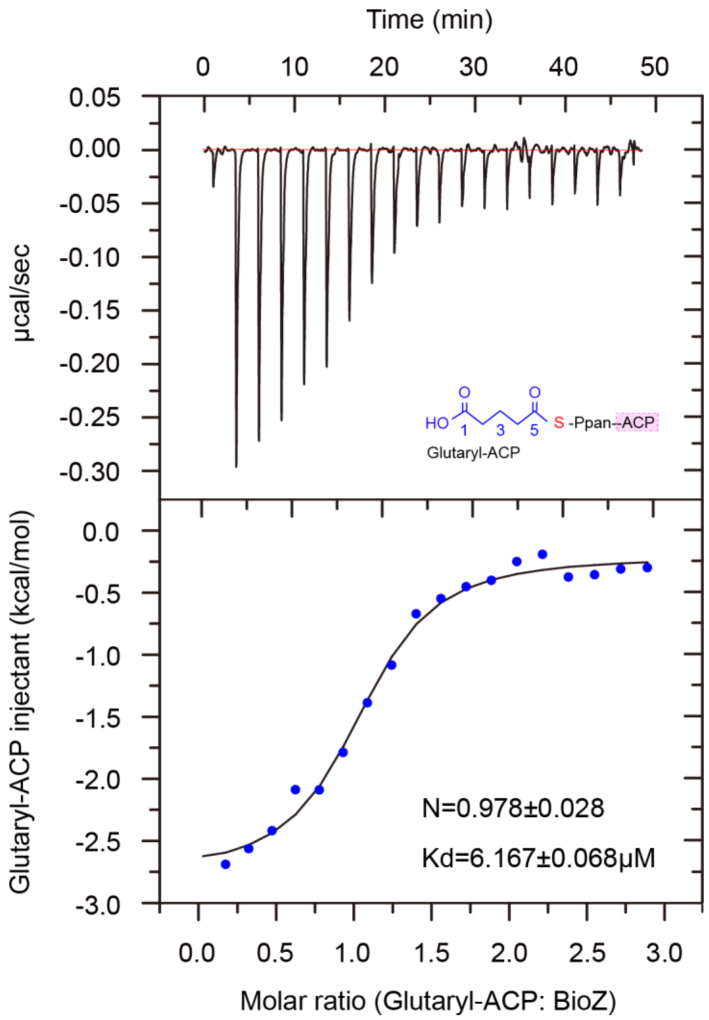

Supplementary Figure 11 Isothermal titration calorimetry (ITC)-based analysis for glutaryl-ACP binding to BioZ protein

A representative result of ITC analyses is given and the stoichiometry value is expressed in an average ± SD.

The molar ratio of Glutaryl-ACP to BioZ was calculated as 0.978, and this validated the stoichiometry of 1:1. The mutant protein of BioZ, BioZ(C115A) was used here to avoid its enzymatic activity in the ITC assay.

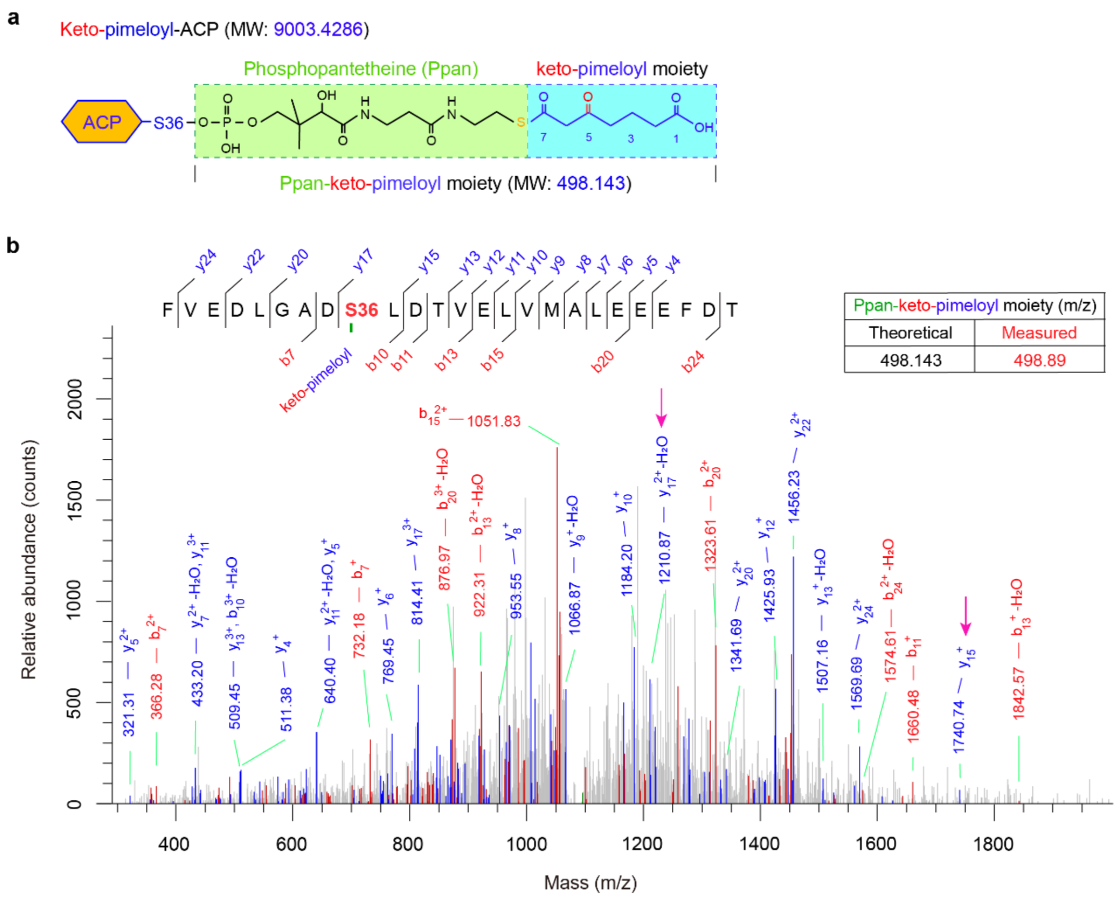

Supplementary Figure 12 Presence of the intermediate of keto-pimeloyl ACP in the *in vitro* BioZ reaction

a. Cartoon scheme for chemical structure of keto-pimeloyl ACP thioester

b. MS/MS determination of keto-pimeloyl ACP

The two peptides used to determine acyl modification were highlighted with pink arrows. The calculated mass was 498.89, which is quite close to the theoretical value (498.143) of Ppan-linked keto-pimeloyl moiety. Thus, it was interpreted as modification of keto-pimeloyl moiety (of note, it might be not stable when compared with pimelate).

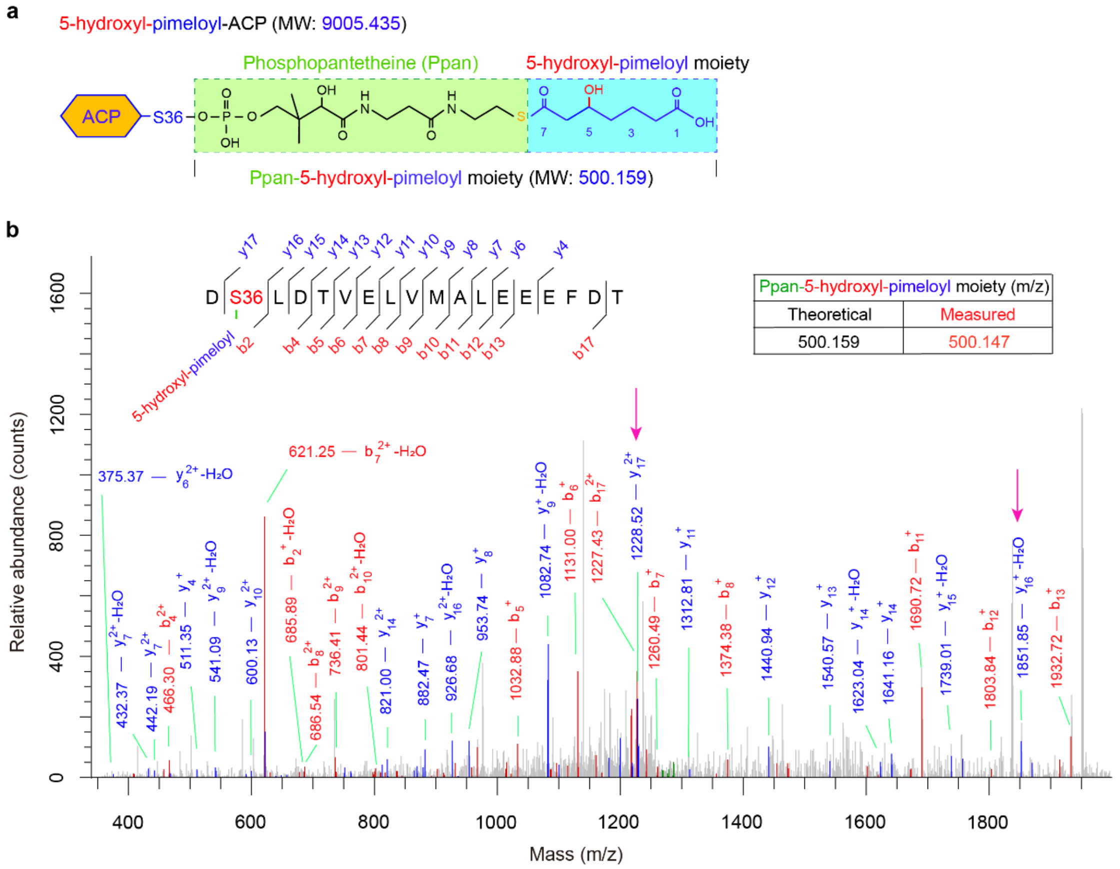

Supplementary Figure 13 Detection of 5-hydroxyl-pimeloyl ACP intermediate from the *in vitro* BioZ reaction

a. Scheme representative for chemical structure of 5-hydroxyl-pimeloyl-ACP thioester

b. Use of high-resolution MS/MS to trap the ACP peptide with the modification of 5-hydroxyl-pimeloyl moiety

The two peaks of peptides labeled with pink arrows are applied in the determination of acyl modification. Given that the calculated mass of 500.147, is almost equivalent to the theoretical value (500.159) of Ppan-linked 5-hydroxyl-pimeloyl moiety, we favored to believe that the modification with hydroxyl-pimeloyl moiety is captured (of note, it might be not as stable as pimelic acid).

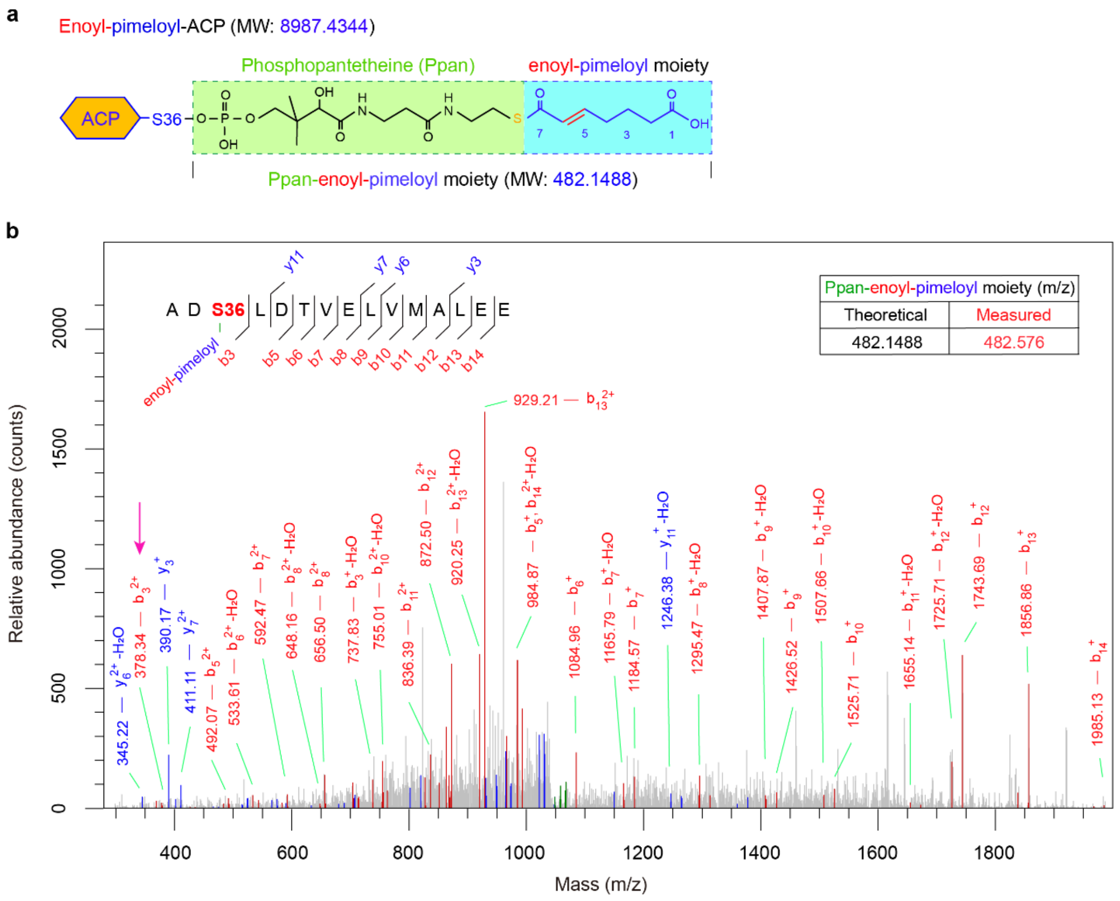

Supplementary Figure 14 Discovery of the enoyl-pimeloyl ACP product from the *in vitro* BioZ reaction

a. Chemical illustration of the enoyl-pimeloyl ACP thioester

b. Use of high-resolution MS/MS to detect the enoyl-pimeloyl ACP peptide fragment

The two peaks of peptides indicated with pink arrows were utilized to determine C7 acyl modification. The measured mass is 482.576, quite close to the theoretical value (482.1488) of Ppan-linked enoyl-pimeloyl moiety. Thus, the modification tracked here refers to enoyl-pimeloyl moiety (of note, it might be not as stable as pimelic acid).

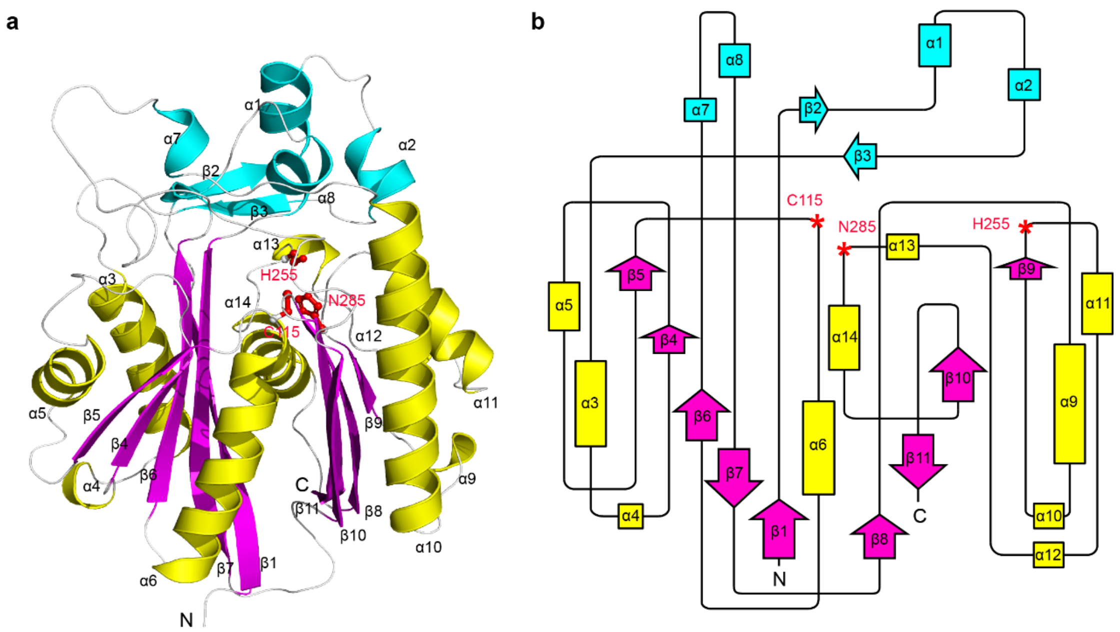

Supplementary Figure 15 Structural characterization of the *A. tumefaciens* BioZ

a. Overall ribbon structure of AtBioZ

b. Topological illustration of AtBioZ

α-helices are colored cyan in top lid domain, whereas it is highlighted with yellow in the bottom core domain. β-sheet is colored magenta, and coils are indicated by grey. The putative three residues (C115, H255, and N285) of catalytic triad are highlighted in ribbon (panel **a**) and labeled with red stars (panel **b**).

Designations: AtBioZ, *A. tumefaciens* BioZ; α, α-helix; β, β-sheet; N, N-terminus; C, C-terminus.

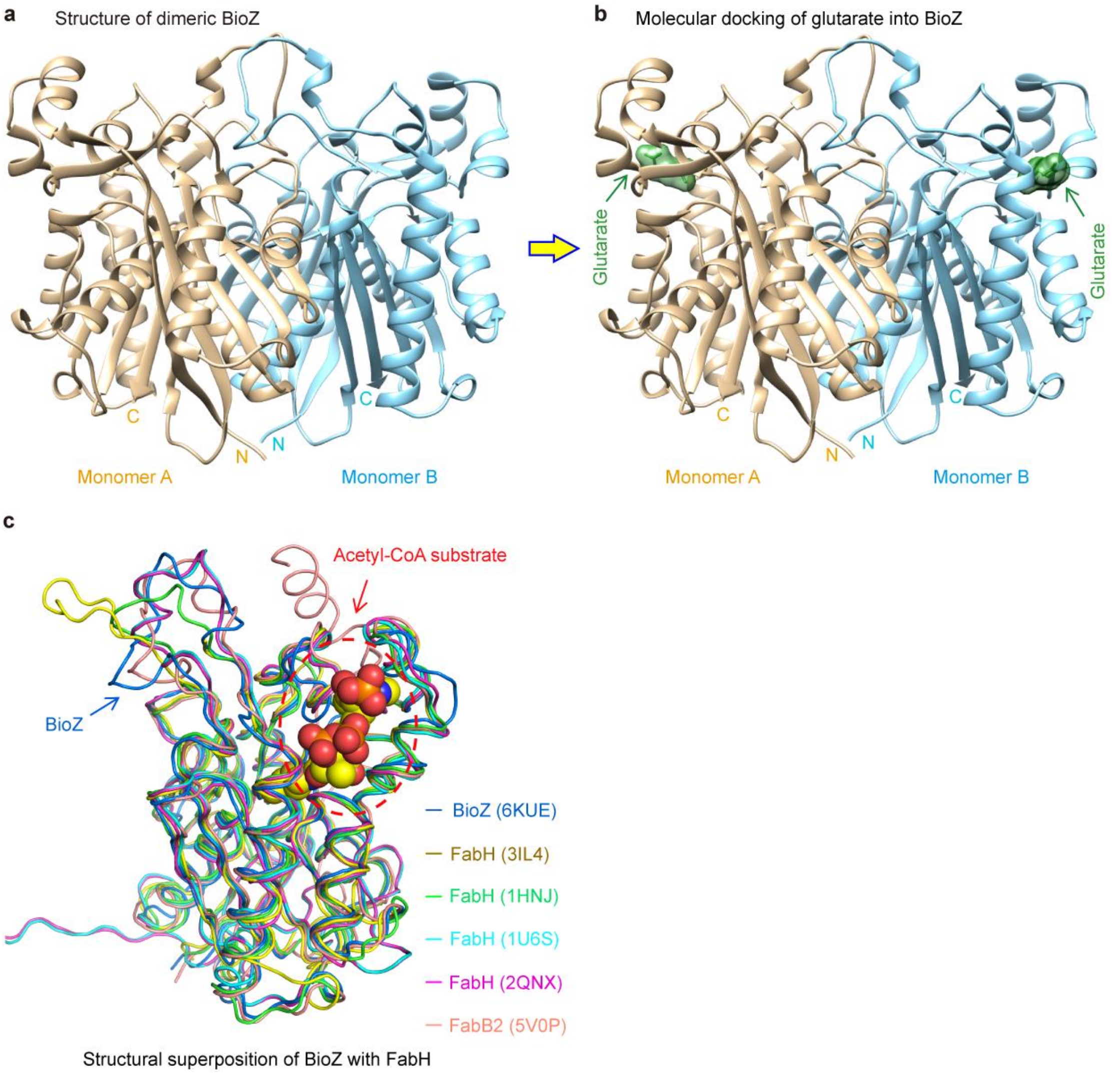

Supplementary Figure 16 Structural illustration of dimeric AtBioZ and its alignment with the ancestor FabH

a. Ribbon structure of AtBioZ in dimer

b. Structure of glutarate-bound AtBioZ revealed by molecular docking

c. Structure of BioZ is superimposed with its ancestor FabH enzymes

Dimeric structure of BioZ is presented, monomer A of which is colored golden, and monomer B is shown in cyan (panel **a**). Because that glutarate is structurally similar to the glutaryl moiety of glutaryl-CoA (ACP), a molecule of glutarate (colored green in panel **b**) is docked into each monomeric AtBioZ, along with appropriately-manual adjustment. The grey ribbon structure of BioZ is indicated with an arrow, and the substrate-loading tunnel is underscored with a circle of dashed blue line (in panel **c**).

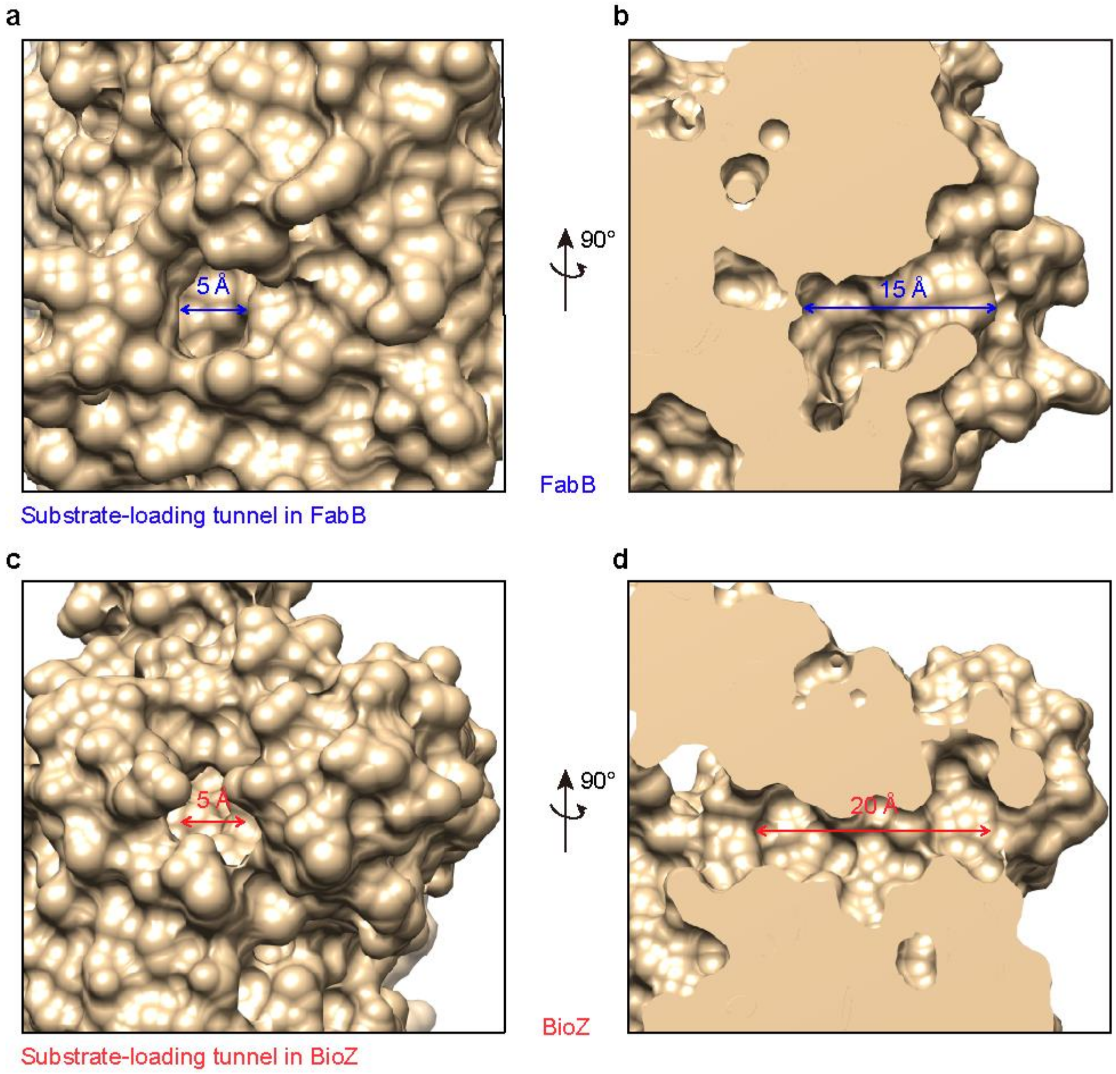

Supplementary Figure 17 Comparative analyses of the substrate-loading tunnel in BioZ and FabB

Surface presentation of the substrate-loading channel in FabB (**a**) and BioZ (**c**)

The entry gate for the substrate-loading channel is measured to be ~5Å in width.

Sectional view of the substrate-loading channel in FabB (**b**) and BioZ (**d**)

It is given via the counter-clockwise 90° rotation, and the depth of tunnel is estimated to be ~15Å for FabB (**b**), and 20Å for BioZ (**d**).

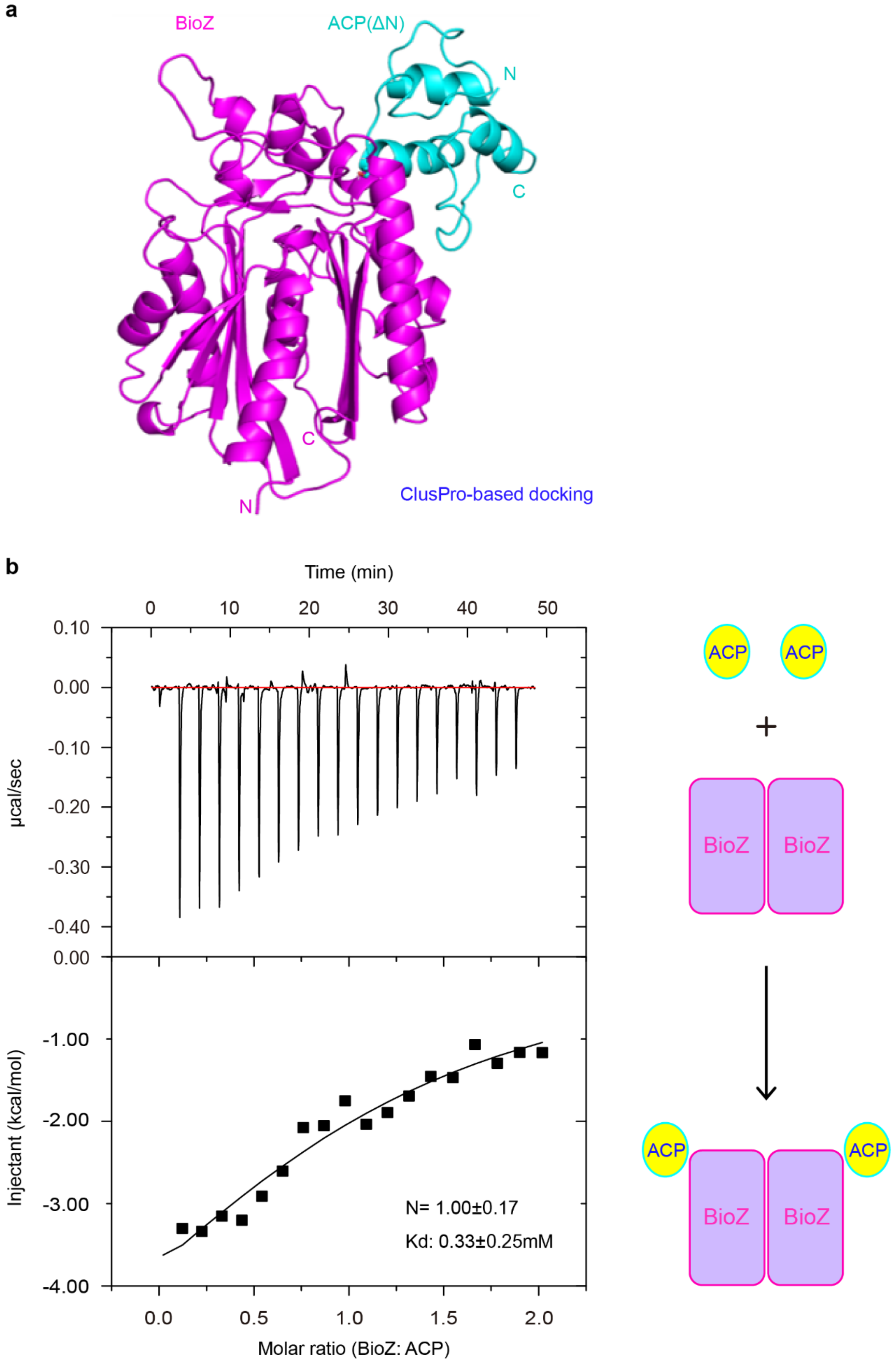

Supplementary Figure 18 Biochemical analysis for an interplay between BioZ and ACP

a. ClusPro-based docking of BioZ with ACP(ΔN), the truncated version of ACP

without a flexible tail at N-terminus
b. ITC measurement of the stoichiometry of BioZ binding ACP

A representative ITC profile is given from three independent measurements and the stoichiometry value is expressed in an average ± SD.

The stoichiometry of BioZ binding to ACP is revealed by ITC to be 1:1.
Kd of ~0.33mM suggests the essence of weak interaction between BioZ and ACP.

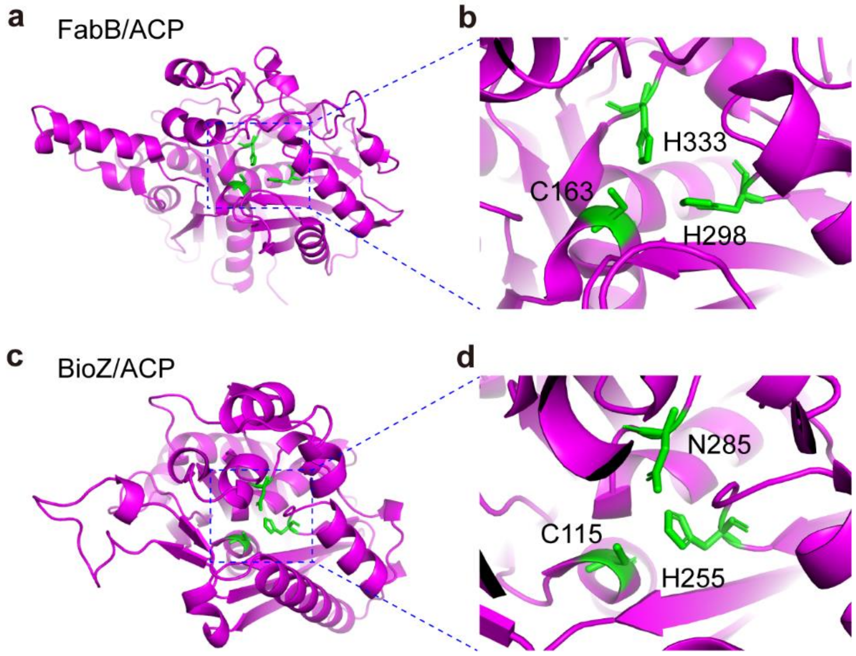

Supplementary Figure 19 Structural insights into substrate recognition and catalysis of BioZ and/or FabB

a-b. Structural analysis of FabB crosslinked with ACP underscores three critical residues (C163, H298 & H333) implicated into the substrate recognition and catalysis

c-d. Structural snapshot of BioZ docked with ACP suggests three potential residues (C115, H255 & N285) involved in the substrate recognition and catalysis

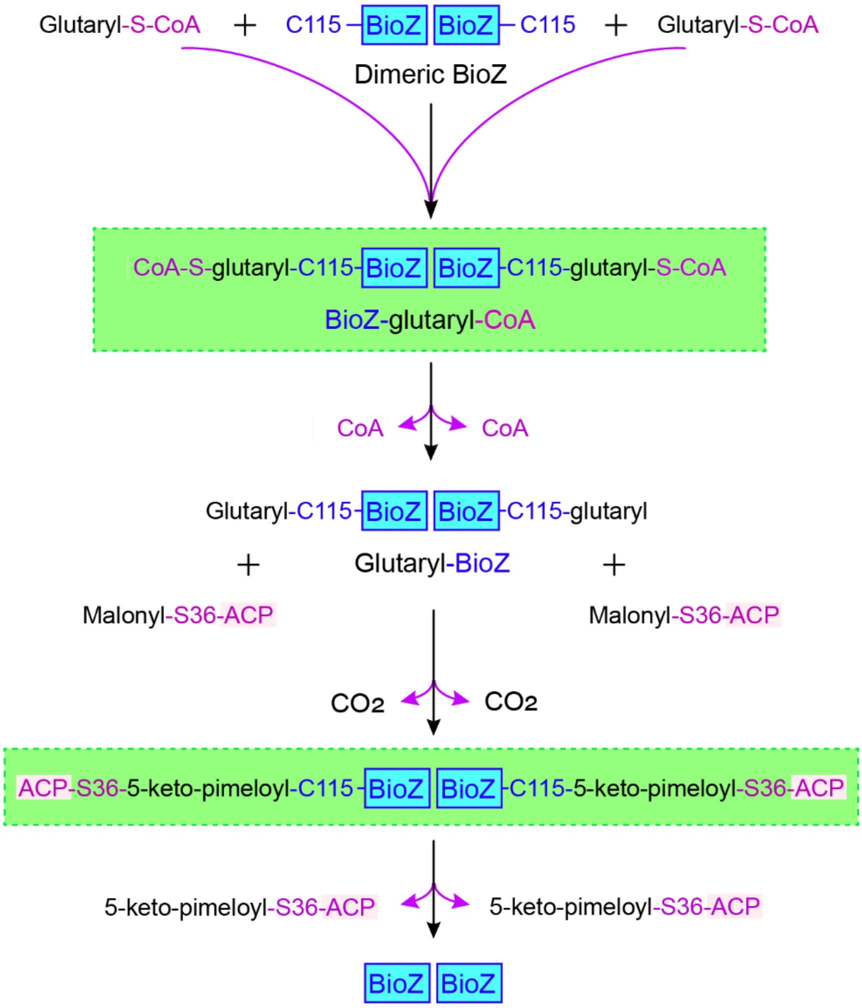

Supplementary Figure 20 A representative scheme for the sequential binding of dimeric BioZ its primer substrate of glutaryl-CoA and then the extension substrate, malonyl-ACP

Given the similarity of both evolutionary origin and structure-to-function assignment occurs amongst FabB, FabF and BioZ, we favored to believe that a sequential mode of BioZ binding to its substrates is reasonable [6,7]. This was also supported by numbers of ITC data.

Supplementary references

1. Feng, Y., Zhang, H. & Cronan, J.E. Profligate biotin synthesis in alpha-proteobacteria - a developing or degenerating regulatory system? *Mol Microbiol* **88**, 77-92 (2013).
2. Lin, S., Hanson, R.E. & Cronan, J.E. Biotin synthesis begins by hijacking the fatty acid synthetic pathway. *Nat Chem Biol* **6**, 682-8 (2010).
3. Wei, W. et al. Molecular basis of BioJ, a unique gatekeeper in bacterial biotin synthesis. *iScience*, DOI:https://doi.org/10.1016/j.isci.2019.08.028 (2019).
4. Jiang, Y., Chan, C.H. & Cronan, J.E. The soluble acyl-acyl carrier protein synthetase of *Vibrio harveyi* B392 is a member of the medium chain acyl-CoA synthetase family. *Biochemistry* **45**, 10008-19 (2006).
5. Feng, Y. et al. A *Francisella* virulence factor catalyses an essential reaction of biotin synthesis. *Mol Microbiol* **91**, 300-314 (2014).
6. Milligan, J.C. et al. Molecular basis for interactions between an acyl carrier protein and a ketosynthase. *Nat Chem Biol* **15**, 669-671 (2019).
7. Mindrebo, J.T. et al. Gating mechanism of elongating beta-ketoacyl-ACP synthases. *Nat Commun* **11**, 1727 (2020).
